# Polypharmacy, potentially serious clinically relevant drug‐drug interactions, and inappropriate medicines in elderly people with type 2 diabetes and their impact on quality of life

**DOI:** 10.1002/prp2.621

**Published:** 2020-07-02

**Authors:** Labib AL‐Musawe, Carla Torre, Jose Pedro Guerreiro, Antonio Teixeira Rodrigues, Joao Filipe Raposo, Helder Mota‐Filipe, Ana Paula Martins

**Affiliations:** ^1^ Faculty of Pharmacy University of Lisbon Lisbon Portugal; ^2^ Centre for Health Evaluation & Research (CEFAR) Lisbon Portugal; ^3^ Nova Medical School Nova University of Lisbon Lisbon Portugal; ^4^ Portuguese Diabetes Association (APDP) Lisbon Portugal

**Keywords:** drug‐drug interactions, elderly, polypharmacy, potentially inappropriate medicines, quality of life, Type 2 diabetes

## Abstract

The aim of the study is to investigate the patterns of polypharmacy, clinical‐relevant drug‐drug interactions (DDIs), and potentially inappropriate medicines (PIMs), and whether polypharmacy, potential serious clinically‐relevant DDIs, or PIMs can be associated with low quality of life (QoL) index scores of older adults with type 2 diabetes (T2D). A cross‐sectional study was conducted using data of 670 elderly T2D sub‐cohort from a nationwide pharmacy‐based intensive monitoring study of inception cohort of T2D in Portugal. 72.09% were found on polypharmacy (≥5 medicines). Participants on polypharmacy were mostly females (*P* = .0115); more obese (*P* = .0131); have more comorbid conditions (*P* < .0001); more diabetes complications (*P* < .0001); and use more of glucose lowering drugs (*P* = .0326); insulin (*P* < .0001); chronic medicines (*P* < .0001); and have higher diabetes duration (*P* = .0088) than those without polypharmacy. 10.59% of the participants were found to have potential serious clinically relevant DDIs. The most frequent drug‐combinations were angiotensin‐converting enzyme (ACE) inhibitors with angiotensin‐receptor blockers (ARBs), aspirin with Selective serotonin reuptake inhibitors (SSRIs), and clopidogrel with calcium channel blockers. PIMs are found in 36.11% of the participants. The most common PIMs were benzodiazepines, long‐acting sulfonylureas, and iron overdose. The adjusted multivariate models show that Polypharmacy, PIMs, and potential serious clinically relevant DDIs were associated with lower QoL index scores (OR 1.80 95% CI 1.15‐2.82), (OR 1.57 95% CI 1.07‐2.28), and (OR 1.34 95% CI 0.73‐2.48) respectively. The study shows that polypharmacy, potential serious clinical‐relevant DDIs, and PIMs may correlate with risk of reduced health related QoL outcome of older adults with T2D.

**What is already known about this subject**

- Polypharmacy is common among the elderly with diabetes.
- Lack of studies addressing the serious clinically relevant drug‐drug interactions (DDIs) and potentially inappropriate medicines (PIMs) in elderly with type 2 diabetes.
- Lack of evidence if polypharmacy and its consequences can impact quality of life (QoL) of elderly with type 2 diabetes.

**What this study adds**

- 72.09% of study cohort are on polypharmacy with poor socio‐demographic profile.
- 10.59% have potentially serious clinically relevant DDIs and 36.11% have PIMs.
- Polypharmacy and its negative consequences may associate with poor QoL.

## 1 INTRODUCTION

The prevalence of elderly people with type 2 diabetes (T2D) has been increasing globally. In 2018, it was estimated that there were more than 500 million people diagnosed with T2D,^1^ and more than half were elderly.^2^ Elderly people with T2D are at higher risk of polypharmacy as result of multimoridity and aging.^3^

Polypharmacy can be associated with several unintended therapeutic outcomes such as increasing the incidence of potential serious drug‐drug interactions (DDIs) that can be harmful and life‐threatening and use of potentially inappropriate medicines (PIMs).^4^, ^5^, ^6^, ^7^

Despite that, there is a paucity in addressing the risk of potential clinically relevant serious DDIs and PIMs. Only one study found that at least one potential serious clinically relevant DDIs was found (7.10%),^8^ and two studies found that the prevalence of PIMs was found between (22.70%‐68.10%).^9^, ^10^ Moreover, there is a lack of evidence on whether the presence of polypharmacy and its consequences can impact quality of life (QoL).

Therefore, the aims of this study was to investigate the patterns of polypharmacy, clinical‐relevant DDIs, and PIMs, and whether polypharmacy, potential serious clinically‐relevant DDIs or PIMs can be associated with low QoL index scores of older adults with T2D.

## 2 METHODS

A cross‐sectional study was conducted using the baseline data of elderly (aged 65 years or more) cohort from a nationwide pharmacy‐based intensive monitoring study of inception cohort of T2D patients using the recently launched glucose lowering drugs (GLDs). Pharmacists and participants recruitment procedures have been described in detail elsewhere.^11^

Invitation letters were sent to all pharmacies from the National Association of Pharmacies that satisfied the inclusion criteria. The pharmacists who agreed to participate were invited to attend a training session in which the study was explained.

The eligible study population consisted of first users of the new GLD (defined as users who did not take the inception‐monitored drug within the 6 months prior to recruitment, as self‐reported by the patients) that were reimbursed in Portugal at the time of enrollment: dipeptidyl peptidase‐4 inhibitor (DPP‐4) alone or in fixed‐dose combination with metformin, glucagon like peptide 1 receptor agonists (GLP‐1 ra), or sodium‐glucose transport protein 2 (SLGT‐2). In this context, the inception drug corresponded to the GLD within the monitored therapeutic classes (DPP‐4, GLP‐1 ra, or SLGT‐2) which the patient was identified with at cohort entry.

The cohort was divided into two subgroups according to participants’ T2D treatment experience: incident new users; participants who were using one of the monitored drugs for the first time and had no current or prior experience with DPP‐4, GLP‐1 ra, or SGLT2 and prevalent new users; participants who had previously used or were still using least one drug of the monitored treatment classes: DPP‐4, GLP‐1 ra, or SGLT2, but not the inception GLD.

At recruitment, participants had a structured face‐to‐face interview with a trained pharmacist to collect the sociodemographic data (birth date, gender, highest educational level completed, co‐residence status, and number of people living in the subject's household), anthropometric data (weight and height were measured by pharmacy staff to calculate the body mass index [BMI]) which was categorized as underweight (<18.50 kg/m^2^), normal (18.50‐24.99 kg/m^2^), overweight (25.00‐29.99 kg/m^2^), and obese (≥30 kg/m^2^). Self‐reported data were collected on clinical characteristics (age at time of T2D diagnosis, clinical care setting), T2D treatment, T2D related complications, co‐morbidities, and concomitant therapy.

### 2.1 Data analysis

Study participants were divided into two subgroups according the presence or absence of polypharmacy. Polypharmacy was defined as the use of five or more medicines, which is the most widely accepted definition in the literature.^12^

The medicines used were checked for the DDIs using IBM Micromedex Platform (IBM^®^ Corporation, 2019).^13^ This platform classify them according to their severity as: ***contraindicated***‐the drugs are contraindicated for concurrent use; ***major*** interaction potential life‐threatening and/or requiring medical intervention to minimize or prevent serious adverse effects; ***moderate*** interaction—may result in exacerbation of the patient's condition and/or require an alteration in therapy; and ***minor*** interaction‐would have limited clinical effects, and generally would not require a major alteration in therapy. Micromedex platform also addresses the potential adverse effect of the interaction, mechanism of the interaction, onset of the interaction, rate of scientific evidence (Excellent/Good/Fair/Unknown), and the proposed clinical management of the interaction.

We defined potentially serious clinically relevant DDIs as those having a severity of ***major*** drug‐drug interaction or when the drug combination is ***contraindicated*** with scientific evidence rating of ***excellent*** (defined as controlled studies that have clearly established the existence of the interaction) according to Micromedex.

PIMs were identified using STOPP criteria version 2, the final list included 80 STOPP criteria, was agreed after two rounds of Delphi validation, which was arranged according to the physiological systems of the body for ease of use and rapid application.^14^

In terms of predictive validity, it modestly discriminates for outcomes such as adverse drug events, emergency department visits, and hospital admissions. The STOPP criteria version 2 has a high sensitivity in detecting PIMs and good inter‐rater reliability.^15^, ^16^, ^17^

The QoL was measured using the three‐level EuroQol five‐dimensional (EuroQol 5‐D‐3L) questionnaire. The EQ‐5D encompass five dimensions influencing health (mobility, self‐care, usual activities, pain/discomfort, anxiety/depression) each with three levels of functioning (first level; no problem, second level; some problems, third level; severe problems).

The summary scores was computed to Portuguese preference weighted EQ‐5D index scores using Portuguese values set.^18^ After that, the study participants finished the EQ‐5D visual analogue scale (VAS). In the VAS, the patients evaluated their current health state on scale between **zero** (worst possible health state) to **one hundred** (best possible health state), the high scores index together with high VAS suggest best health state.^19^

### 2.2 Statistical analysis

A database was created including information on sociodemographic characteristics, comorbidities, and prescribed medicines including both T2D and other chronic medicines, potential (contraindication, serious, moderate, and minor) DDIs, and PIMs. Data were described as absolute and relative counts and means (± SD).

A multivariate binary logistic regression model was used to assess the adjusted associations between polypharmacy, potential serious clinically relevant DDIs, PIMs, and lower QoL scores. Based on Portuguese elderly population preferences, mean index score of QoL was considered (0.60) as cut‐off value.^20^ Results of this analysis were presented as adjusted odds ratios (ORs) and their respective 95% confidence intervals (CIs). Data analysis was performed using SAS® software.

## 3 RESULTS

### 3.1 Characteristics of study population

Of the 1328 adults with T2D recruited in the original cohort, 670 were elderly people with T2D included in the current study. Of these, 483 (72.09%) were on polypharmacy. Among those on polypharmacy, 75.57% (n = 365) and 24.43% (n = 118) were using 5‐9 and ≥10 different medicines respectively.

Participants on polypharmacy were significantly more females (*P* = .0115), more obese (*P* = .0131), had a higher duration of diabetes (*P* = .0088), more comorbid conditions (*P* < .0001), more diabetes complications (*P* < .0001), using more GLDs treatment (*P* = .0326), insulin use (*P* < .0001), and more chronic medicines (*P* < .0001) compared to those without polypharmacy (Table 1).

**TABLE 1:** Descriptive characteristics of study population according to polypharmacy

Characteristics	Total sample (N = 670)	T2DM on Polypharmacy (N = 483)	T2DM Not on Polypharmacy (N = 187)	*P* value
Gender M/F (%)	338/332 (50.45/49.55)	229/254 (47.41/52.59)	109/78 (58.29/41.71)	*P* = .0115
Age (Mean ± SD)	73.01 ± 6.22	73.21 ± 6.22	72.50 ± 6.22	*P* = .2606
65‐74 (%)	432 (64.48)	303 (62.73)	129 (68.99)	
75‐84 (%)	203 (30.30)	152 (31.47)	51 (27.27)	
≥85 (%)	35 (5.22)	28 (5.80)	7 (3.74)	
BMI (%)				*P* = .0131
Underweight (<18.5 kg/m^2^)	2 (0.29)	2 (0.41)	0 (0)	
Normal (18.5‐24.99 kg/m^2^)	108 (16.12)	77 (15.94)	31 (16.58)	
Preobese (25‐29.99 kg/m^2^)	277 (41.34)	185 (38.30)	92 (49.20)	
Obese (≥30 K/m^2^)	265 (39.55)	207 (42.86)	58 (31.02)	
	NR = (18)	NR = (12)	NR = (6)	
Educational level (%)				*P* = .7507
No Education	128 (19.10)	95 (19.67)	33 (17.65)	
Primary (1‐9 y)	425 (63.43)	304 (62.94)	121 (64.71)	
Secondary (10‐12 y)	54 (8.06)	42 (8.70)	12 (6.42)	
Superior (>12 y)	41 (6.12)	29 (6)	12 (6.42)	
	NR = (22)	NR = (13)	NR = (9)	
Occupation (%)				*P* = .9262
Employed	21 (3.13)	16 (3.31)	5 (1.04)	
Unemployed	4 (0.60)	3 (0.62)	1 (0.53)	
Retired	605 (90.29)	434 (89.86)	171 (91.44)	
Domestic	37 (5.52)	28 (5.80)	9 (4.81)	
	NR = (3)	NR = (2)	NR = (1)	
Living alone				*P* = .5906
Yes	135 (20.14)	100 (20.70)	35 (18.71)	
No	531 (79.25)	381 (78.88)	150 (80.21)	
	NR = (4)	NR = (2)	NR = (2)	
Duration of diabetes (%)				*P* = .0088
Less than one year	57 (8.50)	34 (7.04)	23 (12.30)	
≥1‐<3 y	52 (7.76)	32 (6.63)	20 (10.70)	
≥3‐<6 y	85 (12.68)	64 (13.25)	21 (11.23)	
≥6‐<10 y	77 (11.49)	54 (11.18)	23 (12.30)	
≥10 y	348 (51.94)	272 (56.31)	76 (40.64)	
	NR = (44)	NR = (20)	NR = (24)	
Healthcare setting (%)				*P* = .1821
Primary care	469 (70.00)	331 (68.53)	138 (73.80)	
NonPrimary care	201 (30.00)	152 (31.47)	49 (26.20)	
Comorbidities (%)				*P* < .0001
Yes	629 (93.88)	470 (97.31)	159 (85.03)	
No	41 (6.12)	13 (2.69)	28 (14.97)	
Comorbid conditions (%)				
Hypertension	531 (79.25)	409 (84.68)	122 (65.24)	*P* < .0001
Renal failure	72 (10.74)	63 (13.04)	9 (4.81)	*P* = .0200
Heart failure	125 (18.65)	108 (22.36)	17 (3.52)	*P* < .0001
Dyslipidaemia	398 (59.40)	326 (67.49)	72 (14.91)	*P* < .0001
Thyroid gland	24 (3.58)	21 (4.35)	3 (1.60)	*P* = .0865
Respiratory system	25 (3.73)	21 (4.35)	4 (2.14)	*P* = .1760
Digestive system	31 (4.62)	27 (14.44)	4 (2.14)	*P* = .0565
Musculoskeletal system	19 (2.83)	17 (3.52)	2 (1.07)	*P* = .0866
Prostate hyperplasia	21 (3.13); NR = (332)	13 (2.69); NR = (254)	8 (4.28); NR = (78)	*P* = .5539
Neoplasms	23 (3.43)	14 (2.90)	9 (4.81)	*P* = .2222
Depression	11 (1.64)	7 (1.45)	4 (2.14)	*P* = .5286
Hyperuricemia	16 (2.38)	15 (3.11)	1 (0.53)	*P* = .0506
Other	79 (11.79)	67 (13.87)	12 (6.42)	*P* = .0073
Diabetes complications (%)				*P* < .0001
Yes	179 (26.71)	151 (31.26)	28 (14.97)	
No	482 (71.94); NR = (9)	326 (67.49); NR = (6)	156 (83.42); (NR = 3)	
Retinopathy (%)	120 (17.91)	103 (21.33)	17 (9.09)	*P* = .0002
Nephropathy (%)	74 (11.04)	65 (13.46)	9 (4.81)	*P* = .0014
Diabetic Foot (%)	39 (5.82)	35 (7.25)	4 (2.14)	*P* = .0116
Diabetes Medicines (%)				
Oral GLD treatment	670 (100)	483 (100)	187 (100)	*P* = .0326
Insulin	117 (17.46)	106 (21.95)	11 (5.88)	*P* < .0001
Chronic medicines (%)				*P* < .0001
Yes	458 (68.35)	365 (75.57)	93 (49.73)	
No	193 (28.80); NR = (19)	118 (24.43)	75 (40.11); NR = (19)	
Renin‐angiotensin system medicines	458 (68.35)	365 (75.57)	93 (49.73)	*P* < .0001
Beta‐blocking agents	173 (25.28)	161 (33.33)	12 (6.42); NR = (19)	*P* < .0001
Diuretics	172 (25.67)	160 (33.13)	12 (6.42); NR = (19)	*P* < .0001
Calcium channel blockers	144 (21.49)	130 (26.92)	14 (7.49); NR = (19)	*P* < .0001
Lipid lowering medicines	398 (59.40)	343 (71.01)	55 (29.41)	*P* < .0001
Anti‐thrombotic	259 (38.65)	239 (49.48)	20 (10.70); NR = (19)	*P* < .0001
Acid related disorders medicines	212 (31.64)	196 (40.58)	16 (8.56); NR = (19)	*P* < .0001
Psycholeptics	167 (24.92)	153 (31.68)	14 (7.49); NR = (19)	*P* < .0001
Psychoanaleptics	114 (17.01)	102 (21.12)	12 (6.42); NR = (19)	*P* < .0001
Potentially serious clinically relevant drug‐drug interactions	71 (10.59)	70 (14.49)	1 (0.53)	*P* < .00001
Potentially inappropriate medicines	242 (36.11)	219 (45.34)	23 (12.30)	*P* < .00001

Abbreviations: BMI, body mass index; NR, nonrespondents to the questionnaire in the original study; GLD, glucose lowering drugs, these includes: Gliptins (either alone or in combination), GLP‐1 agonists, SGLT2‐inhbitors, or any combination of any two diabetes study medicines.

### 3.2 Identification of potentially serious clinically relevant DDIs

**FIGURE 1:**
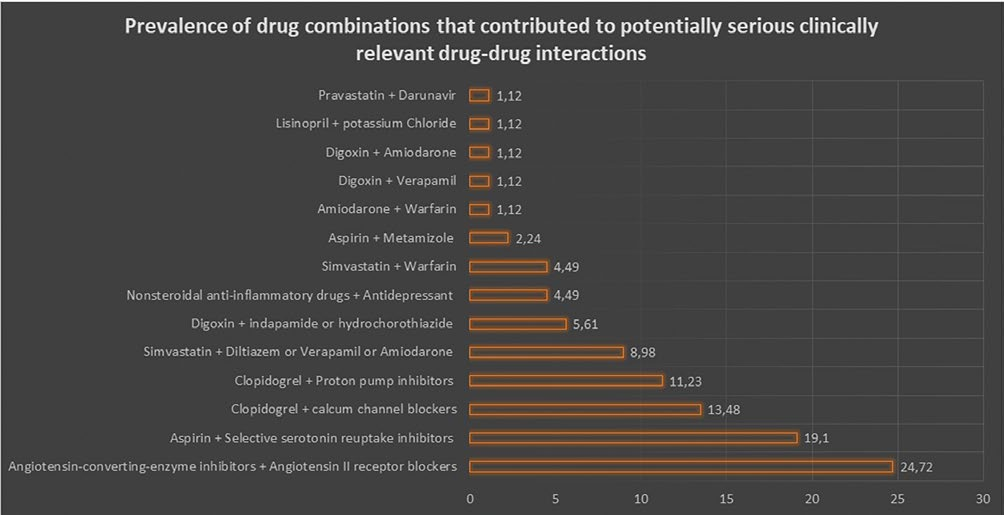
Prevalence of drug combinations that contributed to potentially serious clinically relevant drug‐drug interactions

Of 670 elderly adults with T2D, 71 (10.59% of total cohort) had potentially serious clinically relevant DDIs. Among the most frequent drug‐combinations that contributed to potentially serious clinically relevant DDIs were angiotensin‐converting enzyme (ACE) inhibitors with angiotensin‐receptor blockers (ARBs) (24.71%), aspirin with selective serotonin reuptake inhibitors (SSRIs) (19.10%) and clopidogrel with calcium channel blockers (13.84%; Figure 1). The full description of these DDIs is presented in (Table S2).

### 3.3 Identification of potentially inappropriate medicines

Of the study cohort, 242 (36.11%) had at least one PIMs. Of these, 176 (72.72%) had one PIM, 49 (20.24%) had two PIMs, and 17 had more than two PIMs (7.02%). The mean of PIMs was (1.36 ± 0.78) per patient.

**FIGURE 2:**
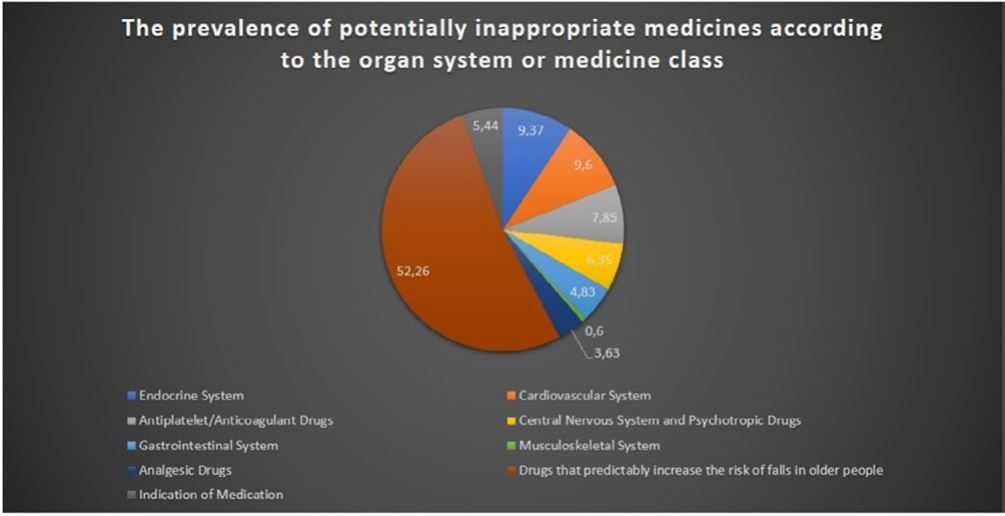
The Prevalence of potentially inappropriate medicines according to the organ system or medicine class

The most prevalent PIMs were benzodiazepines (43.50%), long‐acting sulfonylureas, glibenclamide or glimepiride (9.37%), and higher dose of iron supplements (4.83%; Figure 2). The full description of PIMs is presented in Table S3.

### 3.4 Quality of life

Elderly patients with T2D in the study who were on polypharmacy have some to more severe problems in mobility (*P* = .0004), usual activity (*P* = .0001), personal care (*P* = .0001), pain (0.0007), and anxiety and depression (*P* = .0365), low mean VAS score (63.19 ± 21.24 vs 69.30 ± 19.97, *P* < .0001) and low mean index score (0.58 ± 0.32 vs 0.72 ± 0.24, *P* < .0001), compared with those not on polypharmacy.

The elderly people with T2D with potentially serious clinically relevant DDI have less problems in all EuroQol 5‐D‐3L dimensions, but with low mean VAS score (62.00 ± 20.56 vs 65.16 ± 21.11, *P* = .3466) and low index score (0.54 ± 0.37 vs 0.63 ± 0.29, *P* = .0637) compared with those without potential serious clinically relevant DDIs.

Elderly people with T2D with at least one PIM have some to severe problems in mobility (*P* = .0346), and pain (*P* = .0031), with low mean VAS score (62.32 ± 21.89 vs 66.33 ± 20.45, *P* = .0387) and low mean index score (0.57 ± 0.30 vs 0.65 ± 0.30, *P* = .0003) compared with those without any PIM Table S4.

On the adjusted multivariate analysis, polypharmacy, potential serious clinically relevant DDIs and PIMs were associated with lower index scores (OR 1.80 95% CI 1.15‐2.82), (OR 1.34 95% CI 0.73‐2.48), and (OR 1.57 95% CI 1.07‐2.28) respectively (Table 2).

**TABLE 2:** Results of adjusted multivariate models analyzing polypharmacy with quality of life (QoL), potential serious clinically relevant drug‐drug interactions, and potentially inappropriate medicines with QoL

Model 1			Model 2			Model 3		
Parameter	OR	95% CI	Parameter	OR	95% CI	Parameter	OR	95% CI
Polypharmacy	1.80	1.15‐2.82	Potential serious clinically relevant DDIs	1.34	0.73‐2.48	PIM	1.57	1.07‐2.28
Male	0.47	0.32‐0.68	Male	0.45	0.31‐0.66	Male	0.47	0.33‐0.69
Age (74‐85)	1.63	1.08‐2.47	Age (74‐85)	1.66	1.10‐2.50	Age (74‐85)	1.66	1.10‐2.52
Obesity	1.89	1.09‐3.27	Obesity	1.92	1.11‐3.32	Obesity	1.97	1.14‐3.41
Chronic conditions	3.44	1.24‐9.58	Chronic conditions	4.25	1.56‐11.59	Chronic conditions	4.04	1.47‐11.09
Complications	2.06	1.34‐3.16	Complications	2.14	1.40‐3.28	Complications	2.18	1.42‐3.35

Abbreviation: PIM, potentially inappropriate medicine.

## 4 DISCUSSION

This study show high prevalence of polypharmacy in a cohort of elderly people with T2D when comparing to other countries such as Sweden (56.70%),^21^ Italy (57.10%),^22^ and Greece (22.50%).^23^ This can be explained by a higher overall prevalence of polypharmacy in older population with chronic diseases in Portugal.^24^

Polypharmacy was more prevalent in the elderly women with T2D. This finding was reported in previous studies.^25^, ^26^, ^27^ It can be explained that women tend to be more concerned about their health and seek health services more often.^27^

Obesity was associated with polypharmacy, a finding also in agreement with pre‐existing literature,^22^, ^28^ which could be due to the presence of multimorbid conditions.^28^, ^29^

Duration of diabetes, presence of comorbid conditions and diabetes complications were associated with polypharmacy. T2D itself with wide array of comorbidities such as hypertension, dyslipidaemia, and heart failure, in addition to renal complications can increase the chance of multiple medicines use.^30^

10.59% of the study cohort were found to have potentially serious clinically relevant DDIs, which is considered higher than previously reported (7.10%).^8^ However, a direct comparison is unattainable due to the differences in comorbid conditions and medicines prescribed and different platforms used for assessing DDIs.

These harmful potential interactions may result in increased risk of thrombotic events from decreased antiplatelet effect or bleeding, followed by hypotension or renal failure from cardiovascular medicines, myopathy with statin therapy and increased digoxin concentrations causing risk of toxicity.

Our results were different from previously reported study by *Dumbreck and colleagues* who selected three clinical guidelines produced by the National Institute for Health and Care Excellence (NICE) including T2D, and systematically looked for potentially serious DDIs in relation to another 11 NICE guidelines found that the most common category was cardiovascular related harm such as significant hypotension or bradycardia, followed by increased lithium or digoxin concentrations causing risk of toxicity, myopathy with statin treatment, and renal or serum potassium associated harms.^31^

The most common medicine class combinations involved in potential serious clinically relevant DDIs were ACE inhibitors and ARBs. Prescribers seem to be less aware of the risk from this combination, as it counts for more than (24%) of the total potential serious clinically relevant DDIs.

Both (VALIANT) and (ONTARGET) trials revealed that concurrent use of both ACE inhibitors and ARBs was not associated with reduce the risk of death from cardiovascular causes, myocardial infarction, stroke or hospitalization from heart failure but had significantly increased risk of hypotension, syncope, renal dysfunction, and hyperkalemia, with a trend toward an increased risk of renal dysfunction requiring dialysis.^32^, ^33^

Clopidogrel was the most prevalent interacting medicine involved in potential serious clinically relevant DDIs (24.71%). This can be explained by higher prevalence of heart diseases and use of antiplatelet agents.

Concurrent use of clopidogrel and proton pump inhibitors may be associated with high‐risk of thrombotic events. A recent meta‐analysis found that this combination is associated with increase in composite major adverse cardiac events which is a composite outcome typically comprised of non‐fatal myocardial infarction, non‐fatal stroke, and cardiovascular death (HR 1.28; 95% CI 1.24‐1.32), myocardial infarction (HR 1.51; 95% CI 1.40‐1.62) and stroke (HR 1.46; 95% CI 1.15‐1.86).^34^

Interaction between calcium channel blockers and clopidogrel can be also associated with reduced clopidogrel effect. Nevertheless, there are controversies in the literature, since some studies found a reduction in the effect of clopidogrel with this combination,^35^, ^36^ and other studies could not establish any evidence of reduction in the anti‐platelet activity of clopidogrel.^37^, ^38^

The prevalence of PIMs was found to be 36.11%. This finding is in agreement with previous studies (22.70%‐68.10%).^9^, ^10^ Comparing to the literature, our findings show high prevalence of benzodiazepines use (43.50% vs 5.9%‐14.80%).^9^, ^10^

Benzodiazepines are associated with a higher risk of falls in older adults.^39^ A study conducted in Ireland found that, the use of benzodiazepines was associated with serious falls when coupled with polypharmacy (adjusted relative risk [aRR] 1.40, 95% CI 1.04‐1.87), and associated with a greater number of falls (adjusted incident rate ratio (aIRR) 1.32, 95% CI 1.05‐1.65), independent of polypharmacy.^40^

The use of long‐acting sulfonylureas was the 2nd major PIMs (9.37%) reported. Previous study found that the use of these long‐acting sulfonylureas was associated with increased risk of hip fracture (aOR 1.46, 95% CI 1.17‐1.82) and the risk become higher in those with documented hypoglycemia (aOR 2.42, 95% CI 1.35‐4.34).^41^

The use of higher doses of oral elemental iron was also reported in the study (4.83%), which can be associated with abdominal discomfort, nausea, vomiting, changes in bowel movements, and black stools.^42^

The study revealed that polypharmacy (using 5 or more medicines) was associated with increased risk of low QoL. A study in Spain of elderly population (52.50% of them with T2D) found that the of poor QoL was only associated when polypharmacy defined as the use of 10 or more medicines.^43^

In addition, the study found that the presence of at least one potentially inappropriate medicine, and potential clinically relevant DDIs can be associated with increasing the risk of poor health related QoL in elderly with T2D. To the best of our knowledge, these results have not previously been reported.

Previous study by *Antonio De Vincentis and colleagues* found that only polypharmacy which considered as simple measure surpass PIM and DDI indicators of quality of therapy as it correlate of primary clinical outcomes, that are mortality and rehospitalization^44^

Some limitations were present in the study. Presence of information bias which is characterized by inaccuracy of exact comorbid condition diagnosis and data regarding lab results (eg estimated glomerular filtration rate) were not reported. The data analysed in the present study were baseline data, and we do not know whether the patients really consumed all the dispensed medicines.

The DDIs found in this study were only potential; in other words, no actual outcomes or consequences were evaluated. Finally, due to the nature of the cross‐sectional design, we could not have the opportunity to explore the impact of polypharmacy on symptoms burden or QoL over time.

This study reveals that polypharmacy is common and highly prevalent in cohort of elderly people with T2D, which can be due to disease burden and presence of multimorbid conditions.

The prevalence of potential serious clinically relevant DDIs are relatively low and the medicines concerned are few. The monitoring of patients treated with clopidogrel and other cardiovascular medicines should be improved.

Great attention should be considered while prescribing two different class of cardiovascular medicines with synergism effect that could have potential impact renal function and electrolyte balance, especially in elderly. Precise and updated information on interacting drugs could prevent the occurrence of known interactions, particularly when therapeutic alternatives exist.

Defining the clinical relevance of a DDI is extremely important due to the presence of thousands of theoretically potential DDIs. High‐quality evidence to support the existence of many DDIs is required, which can be established through real‐world observational studies.

STOPP criteria represent the more common avoidable instances of inappropriate prescribing in older people in day‐to‐day clinical practice. Based on our results, risk of fall, fracture or fracture risk, hypoglycemia, and even gastrointestinal side effects can be avoided if prescribers assessed appropriately those elderly patients’ medicines use.

The selection and use of PIM criteria for research or practice should be taken into consideration considering the circumstances and requirements for each case as the relationships with outcomes can be different substantially between tools ^45^

One of the challenges facing healthcare professionals is that the actual harms of both DDIs and PIMs which are poorly quantified in real‐world populations in which people are typically older, frail, have more comorbid conditions and receiving more medicines.

Future studies should have the ability to explore the influence of possible adverse drug events as results of DDIs and PIMs due to polypharmacy on elderly with T2D and the impact on QoL over time in real‐world.

## 5 CONCLUSIONS

The use of polypharmacy is highly prevalent among cohort of elderly people with T2D. This population is at higher risk of potential serious clinically relevant DDIs and PIMs as result of polypharmacy.

The prevalence of potential serious clinically relevant DDIs found is relatively low and can be associated with increased risk of poorer QoL, like polypharmacy and PIMs.

Prospective studies are required to observe the clinical outcomes of the potential serious clinically relevant DDIs and presence of PIMs in real‐world clinical practice. Health Interventions including pharmacist's medication use review and deprescribing strategies may help to improve patient‐centered outcomes.

## 6 AUTHORS' INDIVIDUAL CONTRIBUTIONS

LM, CT, JR, AM, HF, and AR contributed to the design and implementation of the research, LM contributed to the analysis of the results and to the writing of the manuscript with input from all authors, and JG conducted all statistical analyses. All authors reviewed the final manuscript.

## CONFLICT OF INTEREST

All authors declare that they have no conflict of interest.

## Supporting information

Supplementary Material

## Data Availability

The data that support the findings of this study are available from the corresponding author upon reasonable request.
