# Supplementary material for: Polypharmacy, potentially serious clinically relevant drug‐drug interactions, and inappropriate medicines in elderly people with type 2 diabetes and their impact on quality of life
Source: Pharmacol Res Perspect. 2020 Jul 2;8(4):e00621. doi: 10.1002/prp2.621 (PMC7332581; doi:10.1002/prp2.621)
Supplement: Supplementary file 1 — Supplementary Material [file PRP2-8-e00621-s001.doc]

**Polypharmacy, potentially serious clinically relevant drug-drug interactions, and inappropriate medicines in elderly people with type 2 diabetes and their impact on quality of life**

*Labib AL-Musawe*[[1]](#footnote-2)

*Faculty of Pharmacy, University of Lisbon, Lisbon, Portugal*

*Carla Torre*

*Faculty of Pharmacy, University of Lisbon, Lisbon, Portugal*

*Jose Pedro Guerreiro*

*Centre for Health Evaluation & Research (CEFAR), Lisbon, Portugal*

*Antonio Teixeira Rodrigues*

*Centre for Health Evaluation & Research (CEFAR), Lisbon, Portugal*

*Joao Filipe Raposo*

*Nova Medical School, Nova University of Lisbon, Lisbon, Portugal.*

*Portuguese Diabetes Association (APDP), Lisbon, Portugal.*

*Helder Mota-Filipe*

*Faculty of Pharmacy, University of Lisbon, Lisbon, Portugal*

*Ana Paula Martins*

*Faculty of Pharmacy, University of Lisbon, Lisbon, Portugal*

**Supplementary Tables**

**Supplementary Table 1 Description and frequency of potential serious clinically relevant drug-drug interactions**

| **Drug\medicines class** | **Drug\medicines class** | **Potential adverse effect** | **Mechanism** | **Onset** | **N (%)** |
| --- | --- | --- | --- | --- | --- |
| **ACE inhibitors (C09AA)** | **ARBs (C09CA)** | **Risk of Hypotension, syncope, hyperkalemia, changes in renal function, acute renal failure** | **dual blockade of the renin-angiotensin-aldosterone system** | **Out specified** | **22 (24.71)** |
| **Aspirin (B01AC06)** | **Selective serotonin reuptake (SSRIs) inhibitors (N06A)** | **Risk of bleeding** | **depletion of platelet serotonin by SSRI; additive effects** | **Not specified** | **17 (19.10)** |
| **Clopidogrel (B01AC04)** | **Dihydropyridine derivatives (C08CA), SELECTIVE CALCIUM CHANNEL BLOCKERS WITH DIRECT CARDIAC EFFECTS (C08D)** | **Risk of decreased antiplatelet effect and increased risk of thrombotic events** | **inhibition of CYP3A-mediated clopidogrel activation** | **Not specified** | **12 (13.48)** |
| **Clopidogrel (B01AC04)** | **Proton pump inhibitors (A02BC)** | **Risk of reduced antiplatelet activity** | **decreased inhibition of platelet aggregation of clopidogrel by PPIs** | **Rapid** | **10 (11.23)** |
| **Simvastatin (C10AA01)** | **Diltiazem (C08DB01), Verapamil (C08DA01) Amiodarone (C01BD01)** | **increased risk of myopathy, including rhabdomyolysis** | **inhibition of CYP3A4-mediated simvastatin metabolism** | **Rapid** | **8 (8.98)** |
| **Digoxin (C01AA05)** | **Hydrochlorothiazide (C03AA03), indapamide (C03BA11)** | **Risk of digitalis toxicity (nausea, vomiting, arrhythmias)** | **diuretic-induced hypokalemia and hypomagnesemia enhance Na-K-ATPase inhibition by cardiac glycosides** | **Delayed** | **5 (5.61)** |
| **NSAIDs (M01A)** | **Antidepressant (N06A)** | **increased risk of bleeding** | **Unknow** | **Not specified** | **4 (4.49)** |
| **Simvastatin (C10AA01)** | **Warfarin (B01AA03)** | **increased risk of bleeding and an increased risk of rhabdomyolysis** | **competition for cytochrome P450 3A4-mediated metabolism** | **Delayed** | **4 (4.49)** |
| **Aspirin (B01AC06)** | **Metamizole (N02BB02)** | **Risk of reduced efficacy of aspirin** | **attenuated antiplatelet effect of aspirin** | **Not specified** | **2 (2.24)** |
| **Amiodarone (C01BD01)** | **Warfarin (B01AA03)** | **increased INR and an increased risk of bleeding** | **increased exposure to warfarin** | **Delayed** | **1 (1.12)** |
| **Digoxin (C01AA05)** | **Verapamil (C08DA01)** | **increased serum digoxin concentrations, risk of digitalis toxicity and increased risk of complete heart block** | **inhibition of renal and/or extrarenal digoxin clearance; additive effects on AV node conduction** | **Rapid** | **1 (1.12)** |
| **Digoxin (C01AA05)** | **Amiodarone (C01BD01)** | **result in digoxin toxicity and potentiated effects of amiodarone** | **inhibition of p-glycoprotein by amiodarone, and reduction of digoxin clearance; interference with amiodarone by digoxin** | **Not specified** | **1 (1.12)** |
| **Lisinopril (C09AA03)** | **Potassium chloride (A12BA01)** | **Risk of hyperkalemia** | **lowered aldosterone levels** | **Delayed** | **1 (1.12)** |
| **Pravastatin (C10AA03)** | **Darunavir (J05AE10)** | **increased exposure to pravastatin** | **inhibition of CYP3A-mediated pravastatin metabolism by darunavir** | **Not specified** | **1 (1.12)** |

**1 Selective serotonin reuptake inhibitors; 2 proton pump inhibitors; 3 calcium channel blockers; 4 tricyclic anti-depressants; 5 non-steroidal anti-inflammatory drugs; angiotensin converting enzyme inhibitors; 7 angiotensin receptor blockers**

**Supplementary Table 2 Description and frequency of potentially inappropriate medicines detected in the study using STOPP criteria**

| **Section** | **STOPP Criteria** | **N (%)** |
| --- | --- | --- |
| **Endocrine System** | **Sulphonylureas with a long duration of action with type 2 diabetes mellitus** | **31 (9.37)** |
| **Cardiovascular System** | **Using of Centrally acting antihypertensives** | **15 (4.53)** |
|  | **Amiodarone as first-line antiarrhythmic therapy in supraventricular tachyarrhythmias** | **10 (3.02)** |
|  | **Loop diuretic as first-line treatment for hypertension** | **5 (1.51)** |
|  | **Beta-blocker in combination with verapamil or diltiazem** | **1 (0.30)** |
|  | **Verapamil or diltiazem with NYHA¹ Class III or IV heart failure** | **1 (0.30)** |
| **Antiplatelet/Anticoagulant Drugs** | **Antiplatelet agents with vitamin K antagonist, direct thrombin inhibitor or factor Xa inhibitors in patients with stable coronary, cerebrovascular or peripheral arterial disease** | **2 (0.60)** |
|  | **Long-term aspirin at doses greater than 160mg per day** | **8 (2.42)** |
|  | **NSAID² and vitamin K antagonist, direct thrombin inhibitor or factor Xa inhibitors in combination** | **5 (1.51)** |
|  | **NSAID² with concurrent antiplatelet agent(s) without PPI³ prophylaxis** | **5 (1.51)** |
|  | **The use of Ticlopidine** | **6 (1.81)** |
| **Central Nervous System and Psychotropic Drugs** | **Initiation of Tricyclic Antidepressants (TCAs) as first-line antidepressant treatment** | **12 (3.63)** |
|  | **Use of first-generation antihistamines** | **9 (2.72)** |
| **Gastrointestinal System** | **Oral elemental iron doses greater than 200 mg daily** | **16 (4.83)** |
| **Musculoskeletal System** | **COX-2 selective NSAIDs with concurrent cardiovascular disease** | **2 (0.60)** |
| **Analgesic Drugs** | **Use of oral or transdermal strong opioids** | **12 (3.63)** |
| **Drugs that predictably increase the risk of falls in older people** | **Benzodiazepines** | **144 (43.50)** |
|  | **Hypnotic Z-drugs e.g. zopiclone, zolpidem, zaleplon** | **13 (3.93)** |
|  | **Neuroleptic drugs** | **16 (4.83)** |
| **Indication of Medication** | **Duplication drug class prescription** | **18 (5.44)** |
|  | **Total** | **331** |

1 New York Heart Association Functional Classification; 2 Non-steroidal anti-inflammatory drugs; 3 proton pump inhibitors

**Supplementary Table 3 Descriptive analysis of patients with\without polypharmacy, with\without potential clinically relevant drug interactions and with\without potentially inappropriate medicines according to their EuroQol 5-D-3L**

| **Patient Classification** | **Mobility ¹** | | | **Personal care²** | | | **Usual activity³** | | | **Pain⁴** | | | **Anxiety and depression⁵** | | | **VAS⁶ score (mean ±SD)** | **Index score (mean ± SD)** |
| --- | --- | --- | --- | --- | --- | --- | --- | --- | --- | --- | --- | --- | --- | --- | --- | --- | --- |
|  | **No problem** | **Some problems** | **Severe problems** | **No problem** | **Some problems** | **Severe problems** | **No problem** | **Some problems** | **Severe problems** | **No problem** | **Some problems** | **Severe problems** | **No problem** | **Some problems** | **Severe problems** |
| **Polypharmacy** | **231 (35.59)** | **229 (35.29)** | **9 (1.39)** | **375 (57.78)** | **69 (10.63)** | **25 (3.85)** | **299 (46.07)** | **128 (19.72)** | **42 (6.47)** | **225 (34.72)** | **195 (30.09)** | **48 (7.41)** | **266 (41.24)** | **157 (24.34)** | **44 (6.82)** | **63.19 ± 21.24** | **0.58± 0.32** |
| **no polypharmacy** | **122 (18.80)** | **55 (8.47)** | **3 (0.46)** | **166 (25.58)** | **7 (1.08)** | **7 (1.08)** | **145 (22.34)** | **29 (4.47)** | **6 (0.92)** | **108 (16.67)** | **68 (10.49)** | **4 (0.62)** | **107 (16.59)** | **65 (10.08)** | **6 (0.93)** | **69.30 ± 19.97** | **0.72± 0.24** |
| ***P* value** | **0.0001** | | | **0.0004** | | | **0.0001** | | | **0.0007** | | | **0.0365** | | | **<0.0001** | **<0.0001** |
| **Potentially Serious clinically relevant DDIs** | **29 (4.47)** | **34 (5.24)** | **2 (0.31)** | **50 (7.70)** | **8 (1.23)** | **7 (1.08)** | **39 (6.01)** | **17 (2.62)** | **9 (1.39)** | **32 (4.94)** | **25 (3.86)** | **8 (1.23)** | **38 (5.89)** | **20 (3.10)** | **7 (1.09)** | **62.00 ± 20.56** | **0.54 ± 0.37** |
| **No Potentially serious clinically relevant DDIs** | **324 (49.92)** | **250 (38.52)** | **10 (1.54)** | **491 (75.65)** | **68 (10.48)** | **25 (3.85)** | **405 (62.40)** | **140 (21.57)** | **39 (6.01)** | **301 (46.45)** | **238 (36.73)** | **44 (6.79)** | **335 (51.94)** | **202 (31.32)** | **43 (6.67)** | **65.16 ± 21.11** | **0.63 ± 0.29** |
| ***P* value** | **0.2161** | | | **0.0681** | | | **0.0852** | | | **0.4071** | | | **0.5673** | | | **0.3466** | **0.0657** |
| **PIM** | **112 (17.26)** | **118 (18.18)** | **5 (0.77)** | **187 (28.81)** | **36 (5.55)** | **12 (1.85)** | **147 (22.65)** | **68 (10.48)** | **20 (3.08)** | **101 (15.59)** | **107 (16.51)** | **26 (4.01)** | **129 (20.00)** | **81 (12.56)** | **23 (3.57)** | **62.32 ± 21.89** | **0.57 ± 0.30** |
| **No PIM** | **241 (37.13)** | **166 (25.58)** | **7 (1.08)** | **354 (54.55)** | **40 (6.16)** | **20 (3.08)** | **297 (45.76)** | **89 (13.71)** | **28 (4.31)** | **232 (35.80)** | **156 (24.07)** | **26 (4.01)** | **244 (37.83)** | **141 (21.86)** | **27 (4.19)** | **66.33 ± 20.45** | **0.65 ± 0.30** |
| ***P* value** | **0.0346** | | | **0.0929** | | | **0.0524** | | | **0.0031** | | | **0.2852** | | | **0.0387** | **0.0003** |

1-number of non-respondents = 21, 2-number of non-respondents =21, 3-number of non-respondents for =21, 4-number of non-respondents =22, 5-number of non-respondents=25, 6-number of non-respondents for=88.

1. Corresponding author: Labib AL-Musawe PhD student in Pharmacoepidemiology, Department of social pharmacy, Faculty of pharmacy, University of Lisbon Address: Avenida Prof. Gama Pinto, 1649-003 Lisboa, Portugal | Tel (+351) 21 794 64 00 Fax (+351) 21 794 64 70 Email: [labib.almousawe@gmail.com](mailto:labib.almousawe@gmail.com) [↑](#footnote-ref-2)
